# Dr. C. H. Harris’ Case of Congenital Enlargement of the Parotid Gland

**Published:** 1842-12-10

**Authors:** C. H. Harris

**Affiliations:** Buckingham Co., (Va.)


### MEDICAL EXAMINER.

NEW SERIES.

No. 50.] PHILADELPHIA, DECEMBER 10, 1842. [Vol. I.

### *Case of Congenital Enlargement of the Parotid Gland.*

By C. H. Harris, M. D.

To the Editors of the Medical Examiner.

Gentlemen,—I herewith transmit to you the history of a somewhat rare case, which, should you deem it worthy of a place, you can publish in your journal.

Very respectfully yours,

C. H. Harris.

*Buckingham Co.*, (Va.) December 1st, 1842.

On the second of April last, I was called to see a negro child, the property of Mrs. B——, a lady residing in my neighbourhood. I found the patient (an infant about fourteen days old,) with the parotid gland of the left side very much enlarged. Upon inquiry, I ascertained from the mistress of this child, that this enlargement of the gland was discovered about two hours after its birth. Those, who had been with the child from the time of its birth, thought that the size of the gland was pretty much the same, when seen by me, as it was when first noticed by them. My first impression was, that injury had been done to the parotid during labour, in its passage through the bones of the pelvis. But upon examining the gland, it presented a soft and pleasant feeling, with no signs whatever of inflammation about it; my handling it, evidently, gave the child no pain or uneasiness. This fact, with the knowledge of the labour having been an easy one, and moreover the situation of the gland, dispelled all idea of the enlargement having been caused by any injury it might have sustained in its passage through the bony pelvis. It then occurred to me that perhaps I had drawn a wrong diagnosis—that it might be an enlarged lymphatic, and not the parotid—the one being sometimes mistaken for the other. But upon a more minute examination, I clearly satisfied myself, that it was an enlargement of the parotid. Had it been an enlarged lymphatic, and it could have been ascertained that the parents of this child were scrofulous, the conclusion might have at once been drawn, that the tumour was of a scrofulous nature and congenital; but along with the fact of its not being an enlarged lymphatic, it may also be observed that the mother and father of the child are healthy, and descended from very healthy parents. Not being able to ascertain the cause of this enlargement, my advice was not to interfere with it; and, in a few weeks it entirely disappeared, and has never since reappeared.

The interesting point of this case is that of a child having been born with an enlarged parotid gland; for such would seem to have been the fact. The general health of this child has always been good.

